# Essential role for SphK1/S1P signaling to regulate hypoxia-inducible factor 2α expression and activity in cancer

**DOI:** 10.1038/oncsis.2016.13

**Published:** 2016-03-14

**Authors:** P Bouquerel, C Gstalder, D Müller, J Laurent, L Brizuela, R A Sabbadini, B Malavaud, S Pyronnet, Y Martineau, I Ader, O Cuvillier

**Affiliations:** 1 CNRS, Institut de Pharmacologie et de Biologie Structurale, Toulouse, France; 2 Université de Toulouse, UPS, IPBS, Toulouse, France; 3 Equipe Labellisée Ligue contre le Cancer, Toulouse, France; 4 Laboratoire d'Excellence Toulouse Cancer (TOUCAN), INSERM UMR-1037, Cancer Research Center of Toulouse (CRCT), Université de Toulouse, Toulouse, France; 5 Lpath Inc, San Diego, CA, USA; 6 Institut Universitaire du Cancer Toulouse Oncopôle, Toulouse, France

## Abstract

The sphingosine kinase-1/sphingosine 1-phosphate (SphK1/S1P) signaling pathway has been reported to modulate the expression of the canonical transcription factor hypoxia-inducible HIF-1α in multiple cell lineages. HIF-2α is also frequently overexpressed in solid tumors but its role has been mostly studied in clear cell renal cell carcinoma (ccRCC), the most common form of kidney cancer, where HIF-2α has been established as a driver of a more aggressive disease. In this study, the role of SphK1/S1P signaling with regard to HIF-2α was investigated in various cancer cell models including ccRCC cells. Under hypoxic conditions or in ccRCC lacking a functional von Hippel-Lindau (*VHL*) gene and expressing high levels of HIF-2α, SphK1 activity controls HIF-2α expression and transcriptional activity through a phospholipase D (PLD)-driven mechanism. SphK1 silencing promotes a VHL-independent HIF-2α loss of expression and activity and reduces cell proliferation in ccRCC. Importantly, downregulation of SphK1 is associated with impaired Akt and mTOR signaling in ccRCC. Taking advantage of a monoclonal antibody neutralizing extracellular S1P, we show that inhibition of S1P extracellular signaling blocks HIF-2α accumulation in ccRCC cell lines, an effect mimicked when the S1P transporter Spns2 or the S1P receptor 1 (S1P_1_) is silenced. Here, we report the first evidence that the SphK1/S1P signaling pathway regulates the transcription factor hypoxia-inducible HIF-2α in diverse cancer cell lineages notably ccRCC, where HIF-2α has been established as a driver of a more aggressive disease. These findings demonstrate that SphK1/S1P signaling may act as a canonical regulator of HIF-2α expression in ccRCC, giving support to its inhibition as a therapeutic strategy that could contribute to reduce HIF-2 activity in ccRCC.

## Introduction

The bioactive sphingolipid sphingosine 1-phosphate (S1P) is a critical regulator of multifarious physiological and pathophysiological processes including cancer.^1, 2, 3^ S1P can be formed by the phosphorylation of sphingosine, the backbone of sphingolipids, and by the sphingosine kinase-1 (SphK1) isoform.^4^ S1P is a ligand for five high-affinity G protein-coupled receptors (S1P_1–5_), with specific effects dictated by the expression pattern of S1P receptor subtypes expressed in a particular tissue.^5^ S1P is produced intracellularly and exerts its paracrine or autocrine effects by being secreted by specific transporters such as spinster 2 (Spns2).^6, 7, 8^ Alternative GPCR-independent signaling of S1P also exists^9^ with recent studies establishing direct modulation of intracellular proteins.^10, 11^ In cancer, S1P metabolism is often dysregulated directing attention to the SphK1/S1P signaling pathway as a target for anticancer drug discovery.^12, 13, 14^ SphK1 expression is upregulated in tumors, and high SphK1 expression is correlated with a significant decrease in survival rate in patients with several forms of cancer.^15^ In some cases, as in ccRCC, plasma levels of S1P are substantially elevated compared with healthy control levels.^16^ A number of preclinical studies have established that pharmacological inhibition of SphK1 could be efficacious in decreasing tumor size or sensitize to therapeutics.^17, 18, 19, 20^ Interestingly, the anticancer activity of an anti-S1P monoclonal antibody (sphingomab),^21^ which neutralizes S1P and inhibits its extracellular signaling, provides evidence of the importance of exogenous S1P in mediating tumor growth and metastatic potential,^22, 23, 24^ including murine models of ccRCC.^16^

Hypoxia is a characteristic of solid tumors, and the adaptation of cancer cells to hypoxia is instrumental in the development of aggressive phenotype and associated with a poor prognostic in patients.^25^ At the cellular level, the adaptation to hypoxia is predominantly mediated by the hypoxia-inducible factors (HIFs), consisting of an oxygen-sensitive α-subunit and a constituvely expressed β-subunit, that regulate the expression of target genes promoting angiogenesis, glycolysis, metastasis, increased tumor growth and resistance to treatments.^25^ HIF-1α and HIF-2α are the best-characterized HIF-α subunits.^26^ Although HIF-1α is ubiquitously expressed, HIF-2α has a more limited tissue expression and is particularly detected in highly vascularized organs or hypoxic tissues including kidney epithelial cells.^27^ Despite their extensive sequence similarity and co-expression in various cancer cell types, HIF-1α and HIF-2α have non-overlapping roles in tumor progression.^28^ The distinct roles of HIF-1α and HIF-2α in promoting tumor growth have been mainly defined in von Hippel-Lindau (VHL) disease-associated clear cell renal cell carcinoma (ccRCC),^29^ which results in a constitutive expression of either HIF-1α and HIF-2α or HIF-2α alone, and where the role for HIF-2α as a driver of a more aggressive disease has been firmly established.^30, 31^

We previously identified SphK1/S1P signaling as a new modulator of HIF-1α activity under hypoxia owing to a decreased proteasome degradation of HIF-1α subunit mediated by the Akt/GSK3β pathway in various cancer cell models.^32^ More recently, we reported in a prostate cancer animal model that sphingomab, a monoclonal antibody neutralizing extracellular S1P, could reduce hypoxia and associated vascular network malfunction by interfering with HIF-1 activity thus enhancing delivery and efficacy of docetaxel, the standard chemotherapy.^24^

Here, we report that the SphK1/S1P signaling pathway may act as a canonical regulator of HIF-2α expression in multiple cancer cell lineages (lung, prostate and glioma) as well as in ccRCC cell lines (CAKI-1, A498 and 786-O) representing the sub-groups found in human clinic. Therefore, we suggest that targeting the SphK1/S1P signaling represents a strategy that could potentially be exploited in therapeutic approaches to decrease HIF-2 activity in cancer and more particularly in ccRCC. The humanized version of the anti-S1P mAb, sonepcizumab, is currently in a phase II trial in RCC patients (www.clinicaltrials.gov).

## Results

### SphK1 activity regulates HIF-2α expression under hypoxia in multiple cancer cell lineages

**Figure 1:**
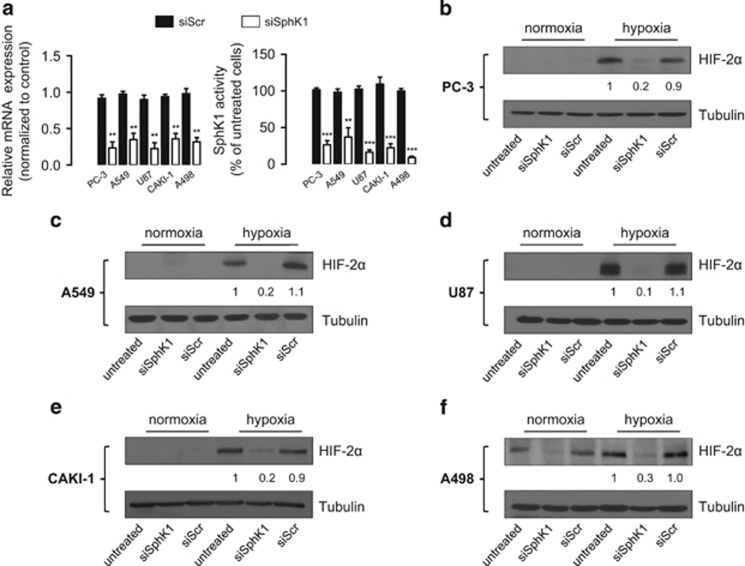
SphK1 silencing prevents HIF-2α accumulation in multiple human cancer cell lines under hypoxia. (**a**), relative mRNA expression of SphK1 expression and SphK1 activity cells were measured in prostate (PC-3), lung (A549), brain (U87) and renal cancer (CAKI-1 and A498) cells after 72 h of treatment with 20 nmol/l of siSphK1 or siScr. *Columns*, mean of at least three independent experiments; *bars*, s.e.m. **P<0.01; ***P<0.001. (**b–f**) PC-3 (**b**), A549 (**c**), U87 (**d**), CAKI-1 (**e**) and A498 (**f**) cells were untreated or treated with 20 nmol/L of siSphK1 or siScr then incubated under normoxia or hypoxia for an additional 6 h. HIF-2α expression was analyzed by immunoblotting. Similar results were obtained in at least three independent experiments, and equal loading was monitored using antibody to tubulin.

To address whether SphK1 has a regulatory role in HIF-2α expression under hypoxia, an siRNA strategy targeting SphK1 (siSphK1) was used. As previously reported,^32^ mRNA level and SphK1 activity were markedly decreased (60–90% range) in all cancer cell lines (prostate PC-3, lung A549, glioblastoma U87, ccRCC CAKI-1 and A498) treated with siSphK1 compared with scrambled siRNA (siScr) (Figure 1a). In all cell lines, hypoxia was associated with a remarkable expression of HIF-2α, which was significantly reduced by siSphK1 treatment (Figures 1b and f), suggesting that SphK1 regulates HIF-2α in addition to HIF-1α as previously published.^32, 33, 34^

Considering the critical role of HIF-2α in ccRCC,^29, 31^ the molecular mechanisms of HIF-2α regulation by SphK1 were further investigated in three ccRCC cell lines, representing the sub-groups found in human clinic (expressing either HIF-1α and HIF-2α or HIF-2α alone). 786-0 and A498 ccRCC cells lack a functional *VHL* gene and express only HIF-2α, whereas CAKI-1 (pVHL wild-type) can produce both HIF-1α and HIF-2α. Noteworthy, in *VHL*-defective A498 where HIF-2α is constitutively present under normoxia due to a lack of degradation, the silencing of SphK1 also repressed its expression (Figure 1f).

### Involvement of Phospholipase D in regulating HIF-2α SphK1-driven expression in ccRCC

**Figure 2:**
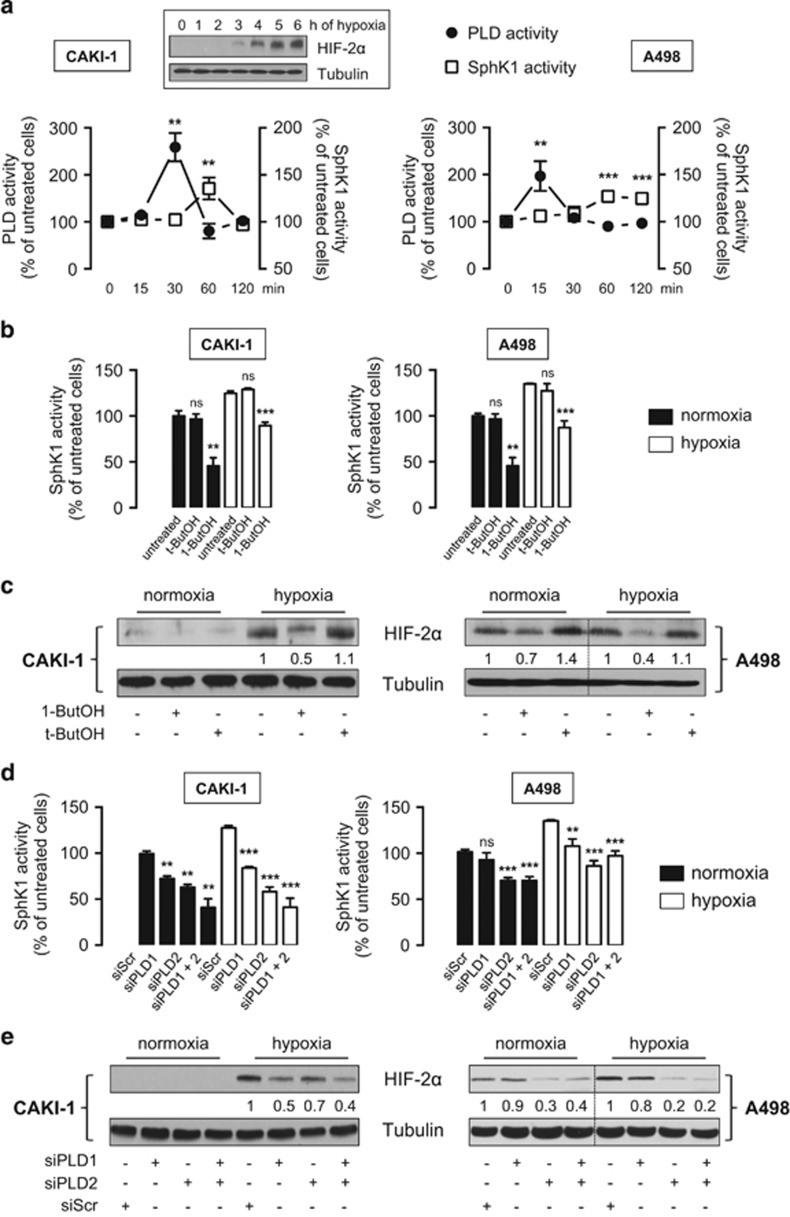
PLD regulates SphK1-dependent HIF-2α expression in CAKI-1 and A498 ccRCC cells. (**a**), CAKI-1 (*left*) and A498 (*right*) cells were incubated under hypoxia for the indicated times and then tested for PLD and SphK1 enzymatic activities. *Points*, mean of at least three experiments; *bars*, s.e.m. **P<0.01; ***P<0.001. Inset, HIF-2α expression in CAKI-1 cells exposed to hypoxia for the indicated times. Similar results were obtained in at least three independent experiments, and equal loading was monitored using antibody to α-tubulin. (**b**, **c**), CAKI-1 (*left*) and A498 (*right*) cells were untreated or treated with 1-butanol (1-ButOH) or tert-butanol (t-ButOH) as control (0.8%). SphK1 activity (**b**) and HIF-2α expression (**c**) were determined in normoxia or after 1 h and 6 h of hypoxia, respectively. Similar results were obtained in at least three independent experiments, and equal loading was monitored using antibody to tubulin. *Columns*, mean of three independent experiments; *bars*, s.e.m. The two-tailed *P-*values between the means of normoxic or hypoxic cells are ns, not significant; **P<0.01; ***P<0.001. (**d**, **e**) CAKI-1 (*left*) and A498 (*right*) cells were transfected with siPLD1 (50 nmol/L), siPLD2 (50 nmol/L) or siPLD1 (50 nmol/L) and siPLD2 (50 nmol/L) or siScr (50 nmol/L) for 72 h then incubated under normoxia or hypoxia. SphK1 activity (**d**) and HIF-2α expression (**e**) were determined after 1 h and 6 h of hypoxia, respectively. Similar results were obtained in at least three independent experiments, and equal loading was monitored using antibody to tubulin. *Columns*, mean of three independent experiments; *bars*, s.e.m. The two-tailed *P*-values between the means of normoxic or hypoxic cells are ns, not significant; **P<0.01; ***P<0.001.

Because phospholipase D (PLD) activity has been involved in the control of HIF-2α expression in ccRCC^35^ and is an upstream regulator of SphK1 in a different physiological context,^36^ we next examined the interactions between PLD and SphK1 signaling with regard to HIF-2α expression. In hypoxic CAKI-1, A498 cells (Figure 2a) and 786-O cells (Supplementary Figure 1A), an early transient increase in PLD activity (peaking at 15–30 min) followed by activation of SphK1 (peaking at 60 min) was observed. Accordingly, accumulation of HIF-2α did not occur before 2–3 h of hypoxia in CAKI-1 cells (Figure 2a, *inset*). Confirming that SphK1 activation was a consequence of PLD stimulation, butan-1-ol (1-ButOH), a potent inhibitor of PLD activation, markedly inhibited SphK1 activity in all ccRCC cell lines (Figure 2b and Supplementary Figure 1B). To rule out any possible non-specific effect of the alcohols on SphK1 activity, cells were treated in the presence of t-butanol (t-ButOH), a tertiary alcohol, which is not a substrate for PLD inhibition. As expected, t-ButOH did not alter SphK1 activity in ccRCC cell lines (Figure 2b and Supplementary Figure 1B). Unlike t-ButOH, 1-ButOH markedly inhibited HIF-2α expression in CAKI-1 cells (Figure 2c, *left*), as well as in normoxic and hypoxic A498 cells (Figure 2c, *right*) and in 786-0 cells (Supplementary Figure 1c). To investigate which PLD isozyme could be involved in the PLD/SphK1/HIF-2α signaling sequence, siRNAs directed to PLD1 and PLD2 isoforms were used.^37^ A roughly 50% PLD knock-down in CAKI-1, A498 and in 786-0 cells was achieved without additive effect when both siRNAs were combined (Supplementary Figure 2). Both PLD1 and PLD2 siRNAs significantly reduced—although not to the same extent—SphK1 activity in CAKI-1 (Figure 2d, *left*), A498 (Figure 2d, *right*) and 786-0 cells (Supplementary Figure 1D). Accordingly, the downregulation of SphK1 activity was accompanied by a marked reduction in HIF-2α expression in hypoxic CAKI-1 cells (Figure 2e, *left*), as well as in normoxic and hypoxic A498 (Figure 2e, *right*) and 786-O cells (Supplementary Figure 1E). These data suggest that both PLD1 and PLD2 isozymes are likely required for regulation of HIF-2α through SphK1 signaling in ccRCC cell lines.

### SphK1 silencing promotes a VHL-independent HIF-2α loss of activity and reduced cell proliferation in ccRCC

**Figure 3:**
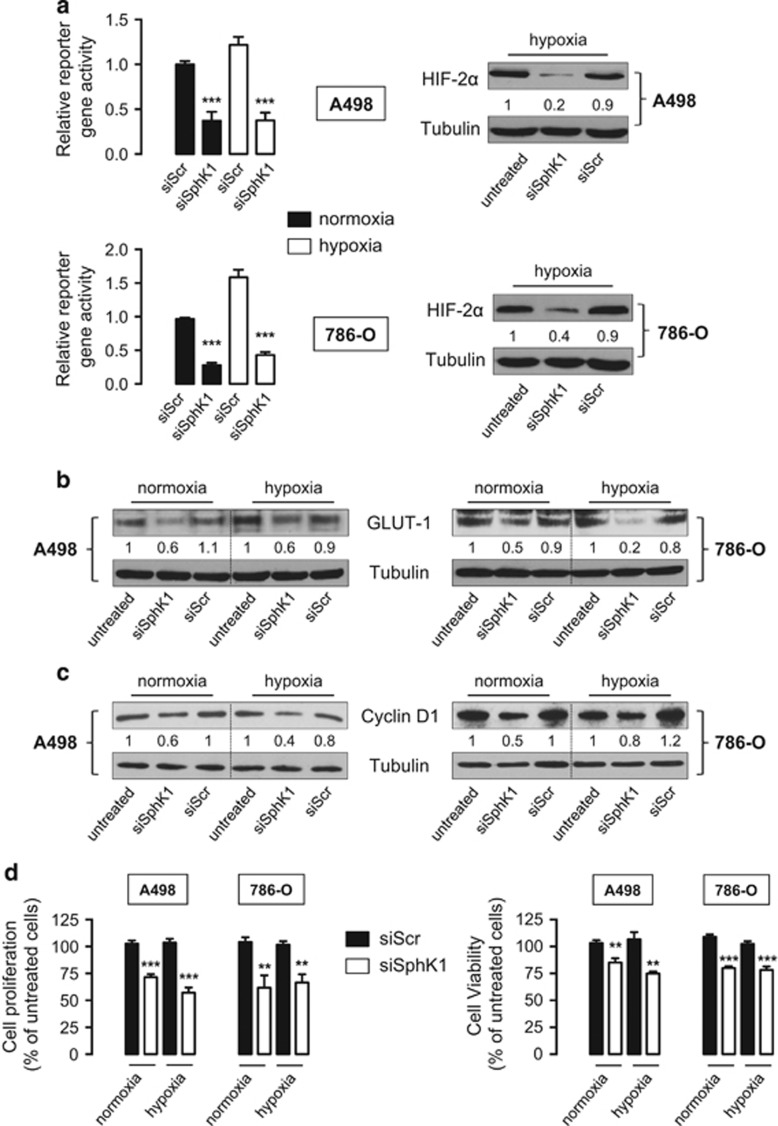
SphK1 silencing leads to a decrease in HIF-2 transcriptional activity in A498 and 786-O *VHL*-defective ccRCC cells. A498 and 786-O cells were treated with 20 nmol/l of siSphK1 or siScr for 72 h then incubated for an additional 16 h under normoxia or hypoxia. (**a**) HRE reporter gene assay (*left*) and protein HIF-2α expression (*right*) in transiently transfected A498 (*upper*) and 786-O (*lower*) cells. The y axis shows normalized *Firefly* luciferase over *Renilla* luciferase activity relative to the wild-type normoxic response. HIF-2α expression was analyzed by immunoblotting. Similar results were obtained in at least three independent experiments, and equal loading was monitored using antibody to tubulin. *Columns*, mean of at least four independent experiments; *bars*, s.e.m. ***P<0.001. Cell lysates were assayed for GLUT-1 (**b**) and cyclin D1 (**c**) expression by western blot analysis. Similar results were obtained in three independent experiments, and equal loading was monitored using antibody to tubulin. (**d**) Cell proliferation and viability was respectively assessed using [^3^H]-thymidine incorporation assay and MTT assay. *Columns*, mean of at least four independent experiments; *bars*, s.e.m. **P<0.01; ***P<0.001.

To establish whether SphK1 silencing was correlated with an inhibition of HIF-2 transcriptional activity, *VHL*-defective A498 and 786-O cells only known to express HIF-2α (vis-à-vis HIF-1α) under either normoxia and hypoxia^38, 39^ were used to avoid the confounding role of HIF-1α we previously reported to be regulated by SphK1 activity.^32^ Accordingly in both *VHL*-defective A498 and 786-O cells, SphK1 silencing was associated with a decreased HIF-2α protein expression (Figure 3a, *right*). The role of HIF-2α transcriptional activity was further investigated by a transient-transfection assay with an HRE reporter gene (pHRE-Luc) for HIF-2α. Accordingly, HRE-mediated transcription was remarkably decreased in A498 and 786-O cells treated with siSphK1 (Figure 3a, *left*) in both normoxia and hypoxia. We next analyzed the level of GLUT-1 and cyclin D1, two well-established specific target proteins of HIF-2α in *VHL*-defective ccRCC.^40, 41^ Under normoxia and hypoxia, SphK1 silencing significantly reduced levels of both glucose transporter GLUT-1 (Figure 3b) and cyclin D1 (Figure 3c) in both cell lines. Cyclin D1 is an important regulator of cell-cycle progression, and *in vitro* and *in vivo* data have demonstrated that HIF-2α-only-expressing ccRCC cells proliferate faster than their HIF-2α and HIF-1α co-expressing counterparts.^42, 43^ Therefore, we next assessed cell proliferation in SphK1-silenced *VHL*-defective ccRCC cells. [^3^H]Thymidine incorporation assay clearly showed that SphK1-silenced A498 and 786-O cells proliferated much slower than their siScr-silenced counterparts (Figure 3d, *left*) and showed a survival disadvantage (Figure 3d, *right*) in both normoxia and hypoxia in line with the findings that HIF-2α likely contributes to tumor cell survival.^39^

### SphK1 silencing is associated with impaired Akt, mTOR, p70S6K and 4E-BP1 phosphorylation in ccRCC

**Figure 4:**
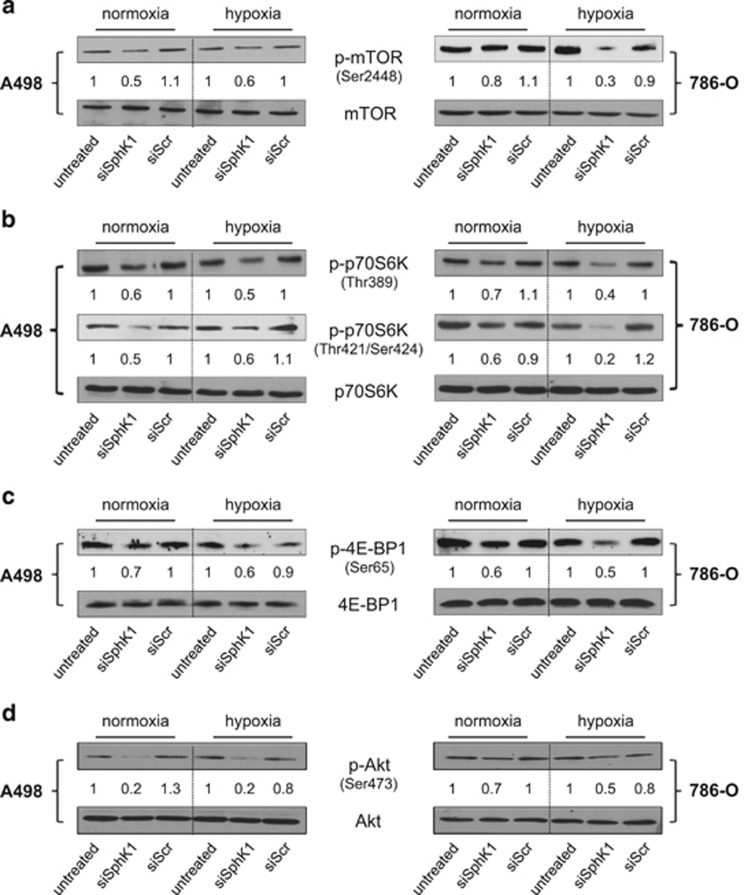
SphK1 silencing downregulates Akt/mTOR signaling pathway in *VHL*-defective A498 and 786-O ccRCC cells. A498 and 786-O cells were untransfected or transfected with 20 nmol/l of siSphK1 or siScr for 72 h before the experiments followed by 4 h under normoxic or hypoxic condition. Cell lysates were assayed for Ser2448 phosphorylated mTOR (p-mTOR Ser2448) and mTOR expression (**a**); Thr389 phosphorylated p70S6K (p-p70S6K Thr389), Thr421/Ser424 phosphorylated p70S6K (p-p70S6K Thr421/Ser424) and p70S6K expression (**b**); Ser65 phosphorylated 4E-BP1 (p-4E-BP1 Ser65) and 4E-BP1 expression (**c**); Ser473 phosphorylated Akt (P-Akt Ser473) and Akt expression (**d**) were analyzed by immunoblotting. Similar results were obtained in three independent experiments.

Akt/mTOR (mammalian target of rapamycin) signaling is often activated in cancer and was previously shown to impact HIF-2α expression.^35^ To explore whether the loss of HIF-2α expression induced by SphK1 silencing was correlated with the inhibition of mTOR signaling, we examined the effect of siSphK1 on levels of total or phosphorylated forms of mTOR (p-mTOR) in A498 and 786-O cells. Under both normoxia and hypoxia, the levels of p-mTOR were clearly reduced after treatment with siSphK1 while total mTOR was not affected (Figure 4a). The regulation of cap-dependent translation initiation by mTOR involves direct phosphorylation of its substrates eukaryotic initiation factor 4E binding protein 1 (4E-BP1) and ribosomal protein kinase S6 (p70S6K).^44, 45^ Activated p70S6K phosphorylates the 40S ribosomal protein S6 whereas phosphorylation of 4E-BP1 disrupts its inhibitory interaction with eukaryotic initiation factor 4E (eIF-4E). SphK1 silencing slightly decreased phosphorylation of p70S6K (Figure 4b) and 4E-BP1 (Figure 4c) in both A498 and 786-O cells.

By activating the tuberous sclerosis complex TSC1/TSC2 leading the activation of mTOR, Akt may represent a mechanistic link between S1P signaling and mTOR signaling as S1P regulates Akt phosphorylation as a ligand for five high-affinity G-coupled receptors (S1P_1-5_) ^46^ in various physiological conditions.^3^ SphK1 silencing in both A498 and 786-O cells was accompanied by a significant reduction in Akt phosphorylation (Figure 4d).

Collectively, these findings suggest that SphK1 silencing reduces the canonical Akt/mTOR pathway.

### SphK1 inhibition does not impact on protein synthesis and stability of HIF-2α in ccRCC

**Figure 5:**
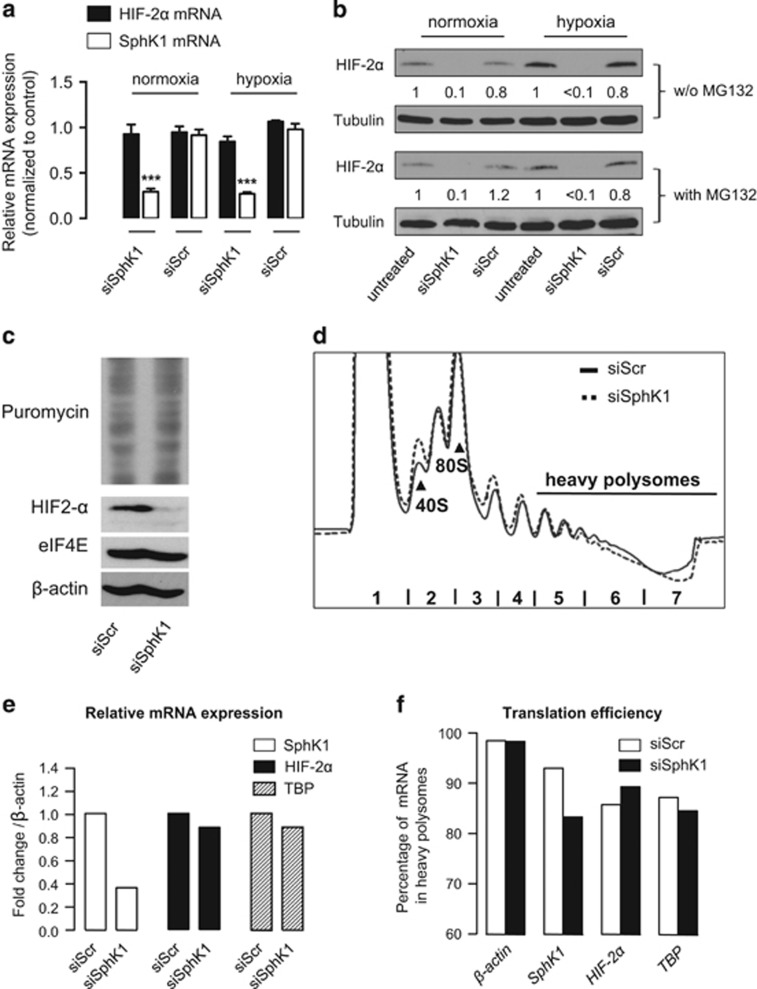
SphK1 signaling does not impact on protein synthesis and stability of HIF-2α in A498 ccRCC cells. (**a**) Relative mRNA expression of HIF-2α and SphK1 expression in A498 cells was measured after 72 h of treatment with 20 nmol/l of siSphK1 or siScr followed by 6 h under normoxic or hypoxic condition. *Columns*, mean of at least four independent experiments; *bars*, s.e.m. ***P<0.001. (**b**) A498 cells were untransfected or transfected with 20 nmol/l of siSphK1 or siScr for 72 h before the experiments. Cells were then incubated for 6 h under normoxic or hypoxic condition in presence or absence of the proteasome inhibitor MG132 (10 μm). Cell lysates were assayed for HIF-2α expression by immunoblotting. Similar results were obtained in three independent experiments, and equal loading was monitored using antibody to tubulin. (**c**) A498 cells were transfected with siSphK1 or siScr as described in Figure 4. 48 h later, cells were treated for 10 min with 10 μg/ml of puromycin, before cells collection. Whole-cell extracts were analyzed by western blot. Puromycin incorporation was measured as readout of protein synthesis using anti-puromycin antibody. (**d**) A498 cells were treated as in (**c**) and subjected to hypotonic lysis. Extracts were separated on 10–45% sucrose gradient and subjected to polysomal profile analysis. 40S ribosomal subunits, 80S ribosomes and heavy polysomes (actively translated mRNA) are indicated. (**e**) RT-qPCR experiments were performed for the indicated mRNAs on RNA extracted from samples in (**d**). Results are presented as fold change normalized to β-actin mRNA and indicate relative mRNA expression. (**f**) RNA was extracted from light fractions (fractions 1–4) and heavy polysomes fractions (fractions 5–7) of the sucrose gradient from (**d**). Relative abundance of the indicated mRNAs in heavy polysomes was quantified by RT-qPCR and presented as translation efficiency for each mRNA. TATA binding protein and β-actin mRNAs were used as controls.

SphK1 signaling could regulate HIF-2α expression and activity at the levels of transcription, translation or protein stability. SphK1 silencing did not alter the mRNA level of HIF-2α in both normoxia and hypoxia in both A498 (Figure 5a) and 786-O (Supplementary Figure 3A) *VHL*-defective ccRCC cells. We next used the proteasome inhibitor MG132 to determine whether the regulatory process mediated by SphK1 was related to proteasome-dependent degradation of HIF-2α. MG132 treatment did not reverse the HIF-2α decrease observed in SphK1-silenced *VHL*-defective ccRCC A498 (Figure 5b) and 786-O cells (Supplementary Figure 3B). Similar findings were observed in *VHL*-positive CAKI-1 ccRCC and A549 lung cancer (Supplementary Figure 3C), suggesting that SphK1 activity regulates HIF-2α content regardless of the presence or absence of VHL.

Following our observation that SphK1 silencing induces a reduced phosphorylation of p70S6K and 4E-BP1 (Figures 4b and c), we analyzed the impact of SphK1 reduction on protein synthesis and accessed the translational regulation of HIF-2α mRNA. Silencing of SphK1 in A498 has no impact on total protein synthesis (Figure 5c) or polysomes formation (Figure 5d), suggesting that the decreased HIF-2α protein abundance is not associated with a global protein synthesis shutdown under normoxic condition. We then measured the translation efficiency of HIF-2α mRNA upon SphK1 silencing. We first confirmed that HIF-2α mRNA abundance remained unchanged on samples used for polysomes fractionation (Figure 5e). We then measured relative abundance of HIF-2α and SphK1 mRNAs in polysomal fractions. TBP and β-actin mRNAs were used as controls. The data indicate that HIF-2α, TBP and β-actin mRNAs relative distribution along polysome fractions remains unchanged upon SphK1 silencing (Supplementary Figure 4) and consequently show no variation of translation efficiency (Figure 5f). In contrast upon siSphK1 transfection, SphK1 mRNA was strongly decreased (70%) at the transcriptional level and was much less translated (10%) as a consequence of a reduced abundance over other mRNAs.

These data indicate that the reduction of HIF-2α protein levels by SphK1 downregulation is not to be due to a translation of HIF-2α mRNA or an enhanced degradation of HIF-2α protein. The slight reduction of 4E-BP1 and p70S6K phosphorylation downstream mTOR observed upon SphK1 silencing was not sufficient to induce a decrease in global protein synthesis, suggesting that other mechanisms might be involved.

### Neutralization of exogenous S1P decreases HIF-2α content in ccRCC

**Figure 6:**
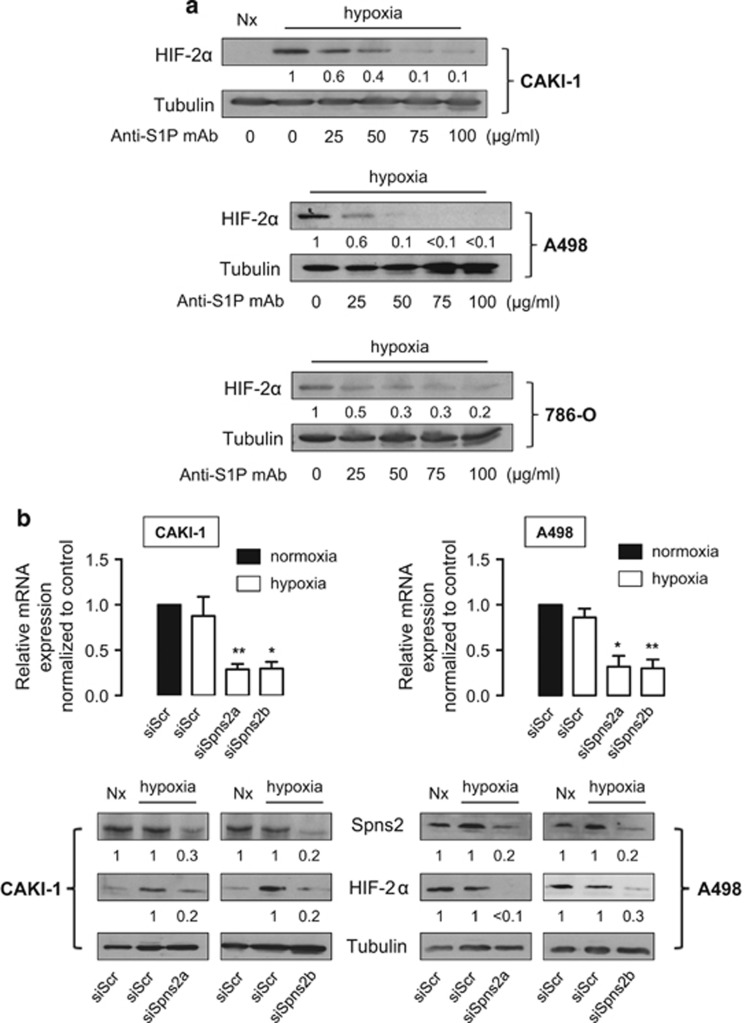
Exogenous S1P regulates HIF-2α accumulation in CAKI-1, A498 and 786-O ccRCC cells. (**a**) CAKI-1, A498 and 786-O cells were treated with the indicated concentrations of anti-S1P mAb for 2 h, then incubated under normoxia or hypoxia for an additional 6 h and HIF-2α expression was analyzed by immunoblotting. (**b**) Relative mRNA expression of Spns2 expression in CAKI-1 and A498 cells was measured after 72 h of treatment with 90 nmol/l of two different siSpns2 (siSpns2a and siSpns2b) or siScr followed by 6 h under normoxic or hypoxic condition. *Columns*, mean of at least four independent experiments; *bars*, s.e.m. *P<0.05; **P<0.01. (**c**) CAKI-1 and A498 cells were transfected with 90 nmol/l of two different siSpns2 (siSpns2a and siSpns2b) or siScr for 72 h, then incubated under normoxia or hypoxia for an additional 6 h. Cell lysates were assayed for Spns2 and HIF-2α expression by immunoblotting. For all experiments, similar results were obtained in at least three independent experiments, and equal loading was monitored using antibody to tubulin.

Owing to the fact that recent studies suggest that exogenous S1P could regulate adaptation to hypoxia through the content of HIF-1α in cancer^24, 34^ and non-cancer cells,^47^ we examined the contribution of extracellular S1P in the regulation of HIF-2α in our ccRCC cell models. We took advantage of the monoclonal antibody sphingomab that binds to and neutralizes extracellular S1P,^21, 22, 23, 24, 48^ which is currently in Phase II clinical trial for metastatic ccRCC. As seen in Figure 6a, sphingomab inhibited HIF-2α protein expression in a concentration-dependent manner in CAKI-1, A498 and 786-O cell lines. This finding is consistent with many reports showing that S1P is produced intracellularly by SphK1 and exerts its paracrine/autocrine effects by being secreted into the tumor microenvironment.^48^ Spinster 2 (Spns2) is believed to be the primary transporter in the release of S1P.^6^ Thus, when CAKI-1 and A498 cells were treated with Spns2-specific siRNAs, the expression of Spns2 protein decreased to less than 20–30% of the control with two different siRNAs (Figure 6b). In line with our recent data suggesting an autocrine effect of S1P in regulating HIF-1α in hypoxic prostate cancer cells,^24^ the silencing of Spns2 was associated with a significant inhibitory effect on HIF-2α accumulation under hypoxia in both CAKI-1 and A498 cells (Figure 6b).

These data establish the exclusive contribution of exogenous S1P mediating the effect of SphK1-driven signaling to regulate HIF-2α content in ccRCC cell lines.

### S1P1 is upregulated under hypoxia and mediates the effect of S1P on HIF-2α

**Figure 7:**
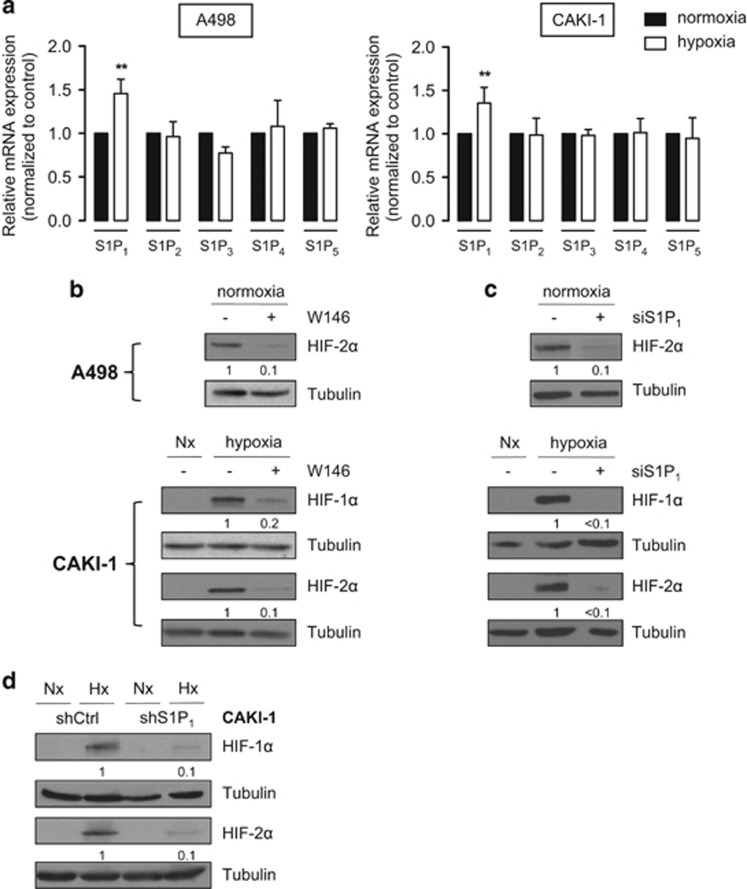
S1P_1_ mediates the effect of S1P on HIF-1α and HIF-2α protein content in CAKI-1 and A498 ccRCC cells. (**a**) The relative mRNA expression of S1P_1–5_ in A498 and CAKI-1 was measured after 1 h of incubation under normoxic (black) or hypoxic (white) conditions. *Columns*, mean of at least five independent experiments; *bars*, s.e.m. **P<0.01. (**b**) A498 and CAKI-1 cells were treated with W146 (5 μm) or ethanol (control), then incubated under normoxia (Nx) or hypoxia for 6 h. HIF-1α and HIF-2α expression was analyzed by immunoblotting. (**c**) A498 and CAKI-1 cells were transfected with 50 nmol/l of siS1P_1_ or siScr for 72 h, then incubated under normoxia (Nx) or hypoxia for an additional 6 h. Cell lysates were assayed for HIF-1α and HIF-2α expression by immunoblotting. (**d**) CAKI-1 shS1P_1_ and CAKI-1 shCtrl cell lines were incubated under normoxia (Nx) or hypoxia (Hx) for 6 h, and HIF-1α and HIF-2α expression was analyzed by immunoblotting. For all experiments, similar results were obtained in at least three independent experiments, and equal loading was monitored using antibody to tubulin.

Exogenous S1P is a ligand for five high-affinity G protein-coupled receptors (S1P_1–5_), with specific effects depending on the suite of S1P receptor subtypes expressed.^5^ Representative CAKI-1 and A498 ccRCC cells express all S1P receptors, we evaluated their involvement in both HIF-1α and HIF-2α regulation. We show that S1P_1_ mRNA is increased after 60 min of hypoxia, while S1P_2–5_ mRNA expression is unchanged (Figure 7a). To investigate the contribution of S1P_1_ in the regulation of HIF-1α and HIF-2α in our models, the targeting of S1P_1_ was achieved with the specific antagonist W146. HIF-2α expression was markedly reduced in both A498 and CAKI-1 cells (Figure 7b). Similarly, the downregulation of S1P_1_ by siRNA strategy in A498 and CAKI-1 cells or shRNA in CAKI-1 cells (Supplementary Figure 5) was accompanied by a strong decrease in HIF-2α expression (Figures 7c and d). We also observed a significant reduction in HIF-1α accumulation under hypoxia in CAKI-1 cells that can produce both HIF-1α and HIF-2α (Figures 7b and c). Similar findings were found in prostate PC-3 and glioblastoma U87 cells (Supplementary Figure 6), suggesting a potential exclusive contribution of S1P_1_ receptor subtype in mediating the effect of S1P on both HIF-1α and HIF-2α content in cancer cells.

## Discussion

HIF-2α is frequently overexpressed in solid tumors,^49^ but its role has been mostly studied in ccRCC, the most common form of kidney cancer, where it has emerged as a key driver in the development and progression of the disease.^50^ A majority of ccRCC (50–80%) exhibit a genetic inactivation of *VHL* gene resulting in the loss of pVHL, which normally mediates ubiquitination of HIF-2α and its subsequent degradation, leading to a constitutive accumulation of HIF-2α.^51^ HIF-2α is both necessary and sufficient to support tumor growth of ccRCC,^52, 53^ whereas the activity of HIF-1α has been shown to be dispensable as its expression is often silenced.^54, 55, 56^ In particular, tumor-promoting genes encoding cyclin D1, TGFα and VEGF have been shown to be driven specifically by HIF-2α,^41^ and HIF-2α-only-expressing ccRCC appears to exhibit a more aggressive clinical behavior.^31^

The relationship between S1P metabolism and HIF signaling has recently emerged in cancer cells from different origins (prostate, thyroid, glioma, lung, kidney and breast) with the majority of studies^32, 33, 34^ establishing that the SphK1/S1P signaling controls the regulation of HIF-1α under hypoxic conditions reviewed in Ader *et al.*^57^ and Cuvillier *et al.,*^58^ whereas an upstream effect of HIF-1α on SphK1/S1P signaling was proposed using a CoCl_2_ chemically-induced model of hypoxia in U87 glioma cells.^59^ With respect to HIF-2α, earlier studies have suggested that HIF-2α transcriptionally upregulates SphK1 expression in CoCl_2_-induced HIF-2α activation in glioma-derived U87 cells.^59^ Although this manuscript was in preparation, Salama *et al.*^60^ reported that HIF-2α was also acting upstream of SphK1/S1P signaling in 786-O ccRCC. As shown in Supplementary Figure 7, we did not find any effect of HIF-2α on SphK1 expression in ccRCC models exhibiting different VHL status (CAKI-1 and A498). In contrast, we show here for the first time that the SphK1/S1P signaling regulates HIF-2α content and activity not only in ccRCC but also in other tumor types (lung, prostate, glioma), suggesting a possible universal regulatory role in cancer cells.

Considering the critical role of HIF-2α in ccRCC pathogenesis, we further detailed the molecular mechanisms of HIF-2α regulation by the SphK1/S1P signaling in various ccRCC models representing the sub-groups found in human clinic,^29^ and by taking advantage of the features of A498 and 786-O *VHL*-defective cells that only express HIF-2α,^38, 39^ and not HIF-1α we previously reported to be regulated by SphK1 signaling.^32^

As previously published for HIF-1α,^32^ a significant rise in SphK1 activity (peaking at 60 min) before HIF-2α accumulation (2–3 h) was observed under hypoxia. PLD activity (peaking at 15–30 min) is known to regulate HIF-2α accumulation in ccRCC,^61^ but a novel finding is that SphK1 is a downstream target of PLD under hypoxia as described earlier in different physiological settings.^36, 62^ Using A498 and 786-O *VHL*-defective cells that only express HIF-2α, we demonstrate that SphK1 activity controls not only HIF-2α protein content but also its transcriptional activity, as downregulation of specific HIF-2-regulated genes such as GLUT-1 or cyclin D1 was observed in SphK1-silenced cells. Accordingly, the proliferation rate and the cell viability were significantly inhibited in SphK1-silenced A498 and 786-O cells, demonstrating that SphK1 activity promotes a survival advantage in accord with the notion that HIF-2α contributes to tumor cell survival.^39^

Importantly, we discovered that SphK1 signaling does not regulate HIF-2α protein content in ccRCC and non-ccRCC cells by a proteasome-dependent mechanism, suggesting a general mode of action regardless of the origin of the cell lines used and the presence or not of pVHL. Rather, SphK1 activity regulates the canonical mTOR pathway, which is often dysregulated in cancer and has a crucial role in the control of HIF-2α translation.^61^

Although, we observed a small decrease in 4E-BP1 and p70S6K phosphorylation, this was not sufficient to induce a marked decrease in global protein synthesis or poly-ribosomes assembly, neither to specifically reduce HIF-2α mRNA translation. Recent studies establish that autophagy might be accountable for HIF-2α protein downregulation in renal cancer.^63^ Sphingolipid-mediated regulation of autophagy exists but the contribution of S1P signaling remains enigmatic and not yet studied in the hypoxia context (reviewed in Li *et al.*^64^). On the basis of our data, we may suspect that SphK1-dependent downregulation of Akt/mTOR pathway might impact the autophagy process leading to the reduction of HIF-2α protein abundance.

We also show that Akt represents a mechanistic link between SphK1/S1P signaling and mTOR signaling, as SphK1 silencing abrogates the Akt/mTOR stimulation. Because Akt signaling can be activated by all Gi-coupled S1P receptor subtypes^5, 65^ and because S1P has been shown to be released from hypoxic cells,^24, 59, 66^ we explored the effects of the neutralization of extracellular S1P with anti-S1P monoclonal antibody sphingomab.^13^ Similar to our recent data showing that sphingomab could block HIF-1α accumulation and activity in prostate cancer cell and animal models,^24^ our findings firmly establish the contribution of extracellular S1P in the regulation of HIF-2α as the silencing of S1P exporter Spns2 as well as the use of sphingomab markedly reduced HIF-2α in all ccRCC subtypes. These data are consistent with our recent report that SphK1 is upregulated in mice xenografts after resistance to VEGFR2 tyrosine kinase inhibition.^16^ Moreover, sphingomab retarded the growth of A498 and 786-O xenografts, particularly after resistance to tyrosine kinase inhibition was established.^16^ In the current work establishing the HIF-2/S1P/hypoxia axis, we also established that S1P_1_ mRNA is upregulated under hypoxia and required for both HIF-1α and HIF-2α accumulation in several human tumor cell lines, while S1P_3_ has previously been shown to be involved in the regulation of HIF-1α in a thyroid cancer model.^34^

In summary, the present report demonstrates that the SphK1/S1P signaling pathway is a potent regulator of HIF-2 in human cancer notably in ccRCC, the most common form of kidney cancer. This disease is aggressive, notoriously resistant to conventional chemotherapy, and is almost invariably an incurable condition.^67^ SphK1/S1P inhibition might represent an alternative therapeutic strategy to current mTOR- or VEGF-targeted agents, as S1P is widely appreciated as a general growth-like factor and a potent protector against apoptosis in addition to its specific regulatory role on HIF-2. The targeting S1P metabolism is ongoing with a humanized version of that anti-S1P mAb, which is being investigated as a treatment for metastatic ccRCC in patients that have failed up to three targeted therapies (www.lpath.com). Anti-S1P strategies could not only provide a beneficial therapeutic option in ccRCC, but also in other cancers as the role of HIF-2 as an oncoprotein might extend beyond renal carcinomas.

## Materials and methods

### Chemicals and reagents

Culture medium and antibiotics were from Lonza (Basel, Switzerland). Serum was from Perbio (Brebières, France). MG132 was obtained from Merck Millipore (Saint-Quentin en Yvelines, France). W146 was from Avanti Polar Lipids (Alabaster, AL, USA). The murine monoclonal anti-S1P antibody, sphingomab, was as described previously.^21^ [γ-^32^P] ATP and [9,10-^3^H(N)]-palmitic acid were from Perkin (Courtaboeuf, France). TLC plates were from VWR (Fontenay sous Bois, France). All other reagents were from Sigma (Saint-Quentin Fallavier, France).

### Cell culture

Human prostate PC-3 and lung A549 cell lines were obtained from DSMZ (Braunschweig, Germany). Human U87 glioblastoma and ccRCC CAKI-1 cells were from ATCC (Molsheim, France). Human ccRCC A498 and 786-O were kindly supplied by Dr G Melillo (NCI, Frederick, MD, USA). CAKI-1 shS1P_1_ and CAKI-1 shCtrl have been established by the Plateau de Vectorologie de Rangueil (CRCT, Toulouse, France) using a pLKO.1-puro-CMV-tGFP plasmid containing a ‘Non Target Control' sequence for shCtrl or a GCTGCTCAAGACCGTAATTAT sequence for shS1P_1_ (clone ID: TRCN0000011360; Sigma). Cells were cultured in RPMI containing 10% FBS at 37 °C in 5% CO_2_ humidified incubators. Cell lines were routinely verified by the following tests: morphology examination, growth analysis and mycoplasma detection (MycoAlert; Lonza). All experiments were started with low-passaged cells (<15 times). Hypoxia (0.1% O_2_, 5% CO_2_, 94.5% N_2_) was achieved using an *In Vivo*2 hypoxic workstation (Ruskinn, Bridgend, UK).

### RNA interference experiments

Transient interference was achieved by double-stranded human siRNAs 5′-GGGCAAGGCCUUGCAGCUCdTdT-3′ (siSphK1) as previously reported;^32, 68^ 5′-AAGGAAACCUAGUAACUGAGC-3′ (siPLD1) and 5′-AAUGGGGCAGGUUACUUUGCU-3′ (siPLD2);^37^ 5′-GGUGGUGUUCAUUCUCAU-3′, 5′-GCUAUAUCACAAUGCUGAA-3′, 5′-GAAGCGCUCUUUACUUGGU-3′ (siS1P_1_, pool);^59^ and MISSION predesigned siRNAs from Sigma for human Spns2: Spns2 siRNAa: 5′-CGCUCAUGCUCUGCCCUUUdTdT-3′ and Spns2 siRNAb: 5′-CACUCAUCCUCAUUCUGGUdTdT-3′.^24^ Aleatory sequence siScr was from Eurogentec (Angers, France). Transfections were carried out using Lipofectamine 2000 in OPTI-MEM medium according to the manufacturer's instructions (Invitrogen, Villebon-sur-Yvette, France).

### SphK1 and PLD enzymatic assays

The protocol for the determination of SphK1 enzymatic activity has been described in details previously.^69^ PLD activity was determined as reported by Brizuela *et al.*^36^ with slight modifications. Cells were treated with the primary alcohol, 1-butanol (0.3%), instead of ethanol (1%). The TLC plates were developed in this case with the superior phase from a mixture of ethylacetate/isooctan/acetic acid/water (55/25/10/50).

### Reporter gene assay

For luciferase assays, cells were plated in 24-well plates and transfected, using Lipofectamine (Invitrogen), with 150 ng of the reporter vector (pRE-tk-LUC) containing three copies of the HRE from the erythropoietin gene and 50 ng of Renilla luciferase plasmid DNA, as an internal control. Twenty-four hours after transfection, cells were incubated under normoxic (20% O_2_) or hypoxic conditions (0.1% O_2_) for 16 h. Cells were then lysed and luciferase activities were measured using the Dual Luciferase Assay Kit (Promega, Charbonnières-les-Bains, France) following the manufacturer's recommendations. Results were quantified with a MicroBeta TRILUX luminescence counter (Perkin Elmer, Villebon-sur-Yvette, France), and normalized values were expressed as the fold induction over control cells.

### Western-blot analysis and antibodies

Rabbit anti-HIF-2α (Novus, Littleton, CO, USA), mouse anti-HIF-1α (BD, San Jose, CA, USA), rabbit anti-GLUT-1 (ThermoScientific, Villebon-sur-Yvette, France), mouse anti-cyclin D1, rabbit anti-p70S6K, rabbit anti-phospho-p70S6K (Thr389), rabbit anti-phospho-p70S6K (Thr421/Ser424), rabbit anti-mTOR, rabbit anti-phospho-mTOR (Ser2448), rabbit anti-4E-BP1, rabbit anti-phospho 4E-BP1 (Ser65), rabbit anti-Akt, rabbit anti-phospho-Akt (Ser473) (Cell Signaling Technology, Danvers, MA, USA), rabbit anti-Spns2 (Sigma), anti-tubulin (Santa Cruz Biotechnology, Santa Cruz, CA, USA), mouse anti-puromycin (Millipore, 12D10) were used as primary antibodies. Proteins were visualized by an ECL detection system (Perbio, Villebon-sur-Yvette, France) using anti-rabbit or anti-mouse horseradish peroxidase-conjugated IgG (Bio-Rad, Marnes-la-Coquette, France). Densitometry quantitation was determined using the Image J software (NIH, Bethesda, MD, USA).

### Quantitative real-time PCR

Total RNA was isolated using the RNeasy minikit (Qiagen, Courtaboeuf, France), and 1 μg was reversed transcribed to cDNA using the SuperScript Frist Stand Synthesis System (Invitrogen). Quantitative real-time PCR was performed using the MESA Blue PCR Master mix (Eurogentec). Reactions were performed using hSphK1-specific primers (forward 5′-CTGGCAGCTTCCTTGAACCAT-3′ reverse, 5′-TGTGCAGAGACAGCAGGTTCA-3′), hHIF-2α-specific primers (forward, 5′-TCCCACCAGCTTCACTCTCT-3′ reverse, 5′-TCAGAAAAAGGCCACTGCTT-3′), hS1P_1_-specific primers (forward, 5′-AAATTCCACCGACCCATGTA-3′ reverse, 5′-AGTTATTGCTCCCGTTGTGG-3′), hS1P_2_-specific primers (forward, 5′-CACCTGGCGGTACAAAGAAT-3′ reverse, 5′-GTCAAGTGGCAGCTGATGAA-3′), hS1P_3_-specific primers (forward, 5′-GCTTCAGGAAATGGAAGCTG-3′ reverse, 5′-TCAGGATGCTGTGAAACTGC-3′), hS1P_4_-specific primers (forward, 5′-CTGCTCTTCACCGCCCTGGC-3′ reverse, 5′-GAAGCCGTAGACGCGGCTGG-3′), hS1P_5_-specific primers (forward, 5′-GTGAGGTGGGAGCCATAGAA-3′ reverse, 5′-TTGGCTGAGTCTCCCAGAGT-3′) and actin-specific primers (forward, 5′-ATTGGCAATGAGCGGTTCC-3′ reverse, 5′-GGTAGTTTCGTGGATGCCACA-3′). β-Actin-specific primers (forward primer, 5′-GCAAAGACCTGTACGCCAAC-3′ reverse primer, 5′-AGTACTTGCGCTCAGGAGGA-3′), TBP-specific primers (forward primer, 5′-TGACCTAAAGACCATTGCACTTCG-3′ reverse primer, 5′-CGTGGTTCGTGGCTCTCTTATC-3′). For analysis, all genes were normalized to expression of zeta polypeptide (*YWHAZ*) gene as an endogenous control. For experiment on translational regulation of HIF-2α mRNA, β-actin and TBP mRNA were used as an endogenous control.

### Sunset assay

The SUnSET assay was used to monitor the rate of protein synthesis as described.^70^ Briefly, 10 min before harvesting the cells, puromycin was added to culture medium at 1 μg/ml. Cell extracts were then processed for western blotting using anti-puromycin antibody.

### Polysome analysis, RNA isolation, microarray and associated RT-qPCR

Cells were washed twice with cold PBS containing 100 μg/ml cycloheximide, collected and lysed in a hypotonic lysis buffer (5 mm Tris-HCl (pH 7.5), 2.5 mm MgCl2, 1.5 mm KCl, 100 μg/ml cycloheximide, 2 mm DTT, 0.5% Triton X-100 and 0.5% sodium deoxycholate). Lysates were loaded onto 10–45% sucrose density gradients (20 mm HEPES-KOH (pH 7.6), 100 mm KCl, 5 mm MgCl_2_) and centrifuged at 45 000 r.p.m. (SW 55 Ti rotor; Beckman Coulter, Villepinte, France) for 45 min at 4 °C. Gradients were fractionated, and the optical density at 254 nm was continuously recorded using an ISCO fractionator (Teledyne ISCO; Lincoln, NE, USA).

Polysome fractions were pooled by two polysome-associated mRNAs, and RNA was isolated using TRIzol-LS (Thermo Fisher, Villebon-sur-Yvette, France). A parallel sample was collected from the postnuclear lysates that were loaded onto the sucrose gradient cytoplasmic mRNA, and RNA was isolated using TRIzol (Thermo Fisher). RT-qPCRs were carried out using RevertAid H Minus Reverse Transcriptase (Thermo Fisher) and SsoFast EvaGreen Supermix (Bio-Rad) according to the manufacturers' instructions.

### Statistical analysis

The statistical significance of differences between the means of two groups was evaluated by unpaired Student's *t*-test. All statistical tests were two-sided, and the level of significance was set at *P<*0.05. Calculations were done using Prism 7 (GraphPad Software, San Diego, CA, USA).
