# Supplementary material for: Essential role for SphK1/S1P signaling to regulate hypoxia-inducible factor 2α expression and activity in cancer
Source: Oncogenesis. 2016 Mar 14;5(3):e209–. doi: 10.1038/oncsis.2016.13 (PMC4815047; doi:10.1038/oncsis.2016.13)
Supplement: Supplementary Figure 1 [file oncsis201613x1.pdf]

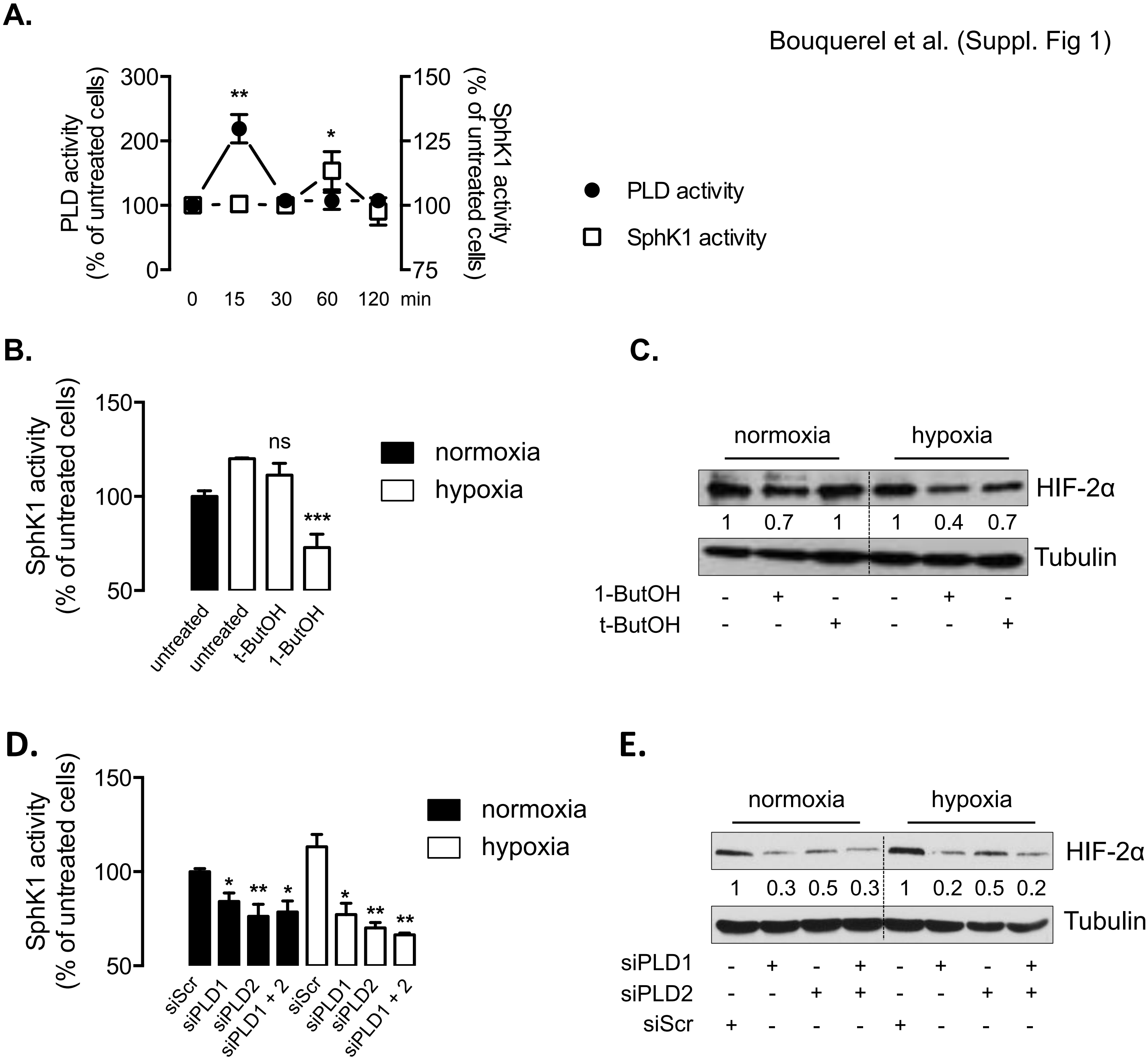

Phospholipase D regulates SphK1-dependent HIF-2α expression in 786-O cells
A, cells were incubated under hypoxia for the indicated times and then tested for PLD and SphK1 enzymatic activities. *Points*, mean of at least three experiments; *bars*, SEM. The two-tailed P values between the means of hypoxic cells are : *, P<0.05; **, P<0.01.
B-C, cells were untreated or treated with 1-butanol (1-ButOH) or tert butanol (t-ButOH) as control (0.8%). SphK1 activity (**B**) and HIF-2α expression (**C**) were determined after 1h and 6h of hypoxia, respectively. Similar results were obtained in at least three independent experiments, and equal loading was monitored using antibody to α-tubulin. *Columns*, mean of three independent experiments; *bars*, SEM. The two-tailed P values between the means of hypoxic cells are : ***, P<0.001.
D-E, cells were transfected with siPLD1 (50 nmol/L), siPLD2 (50 nmol/L) or siPLD1 (50 nmol/L) and siPLD2 (50 nmol/L) or scrambled siRNA (siScr, 50 nmol/L) for 72h then incubated under normoxia or hypoxia. SphK1 activity (**D**) and HIF-2α expression (**E**) were determined after 1h and 6h of hypoxia, respectively. Similar results were obtained in at least three independent experiments, and equal loading was monitored using antibody to α-tubulin. *Columns*, mean of three independent experiments; *bars*, SEM. The two-tailed P values between the means of hypoxic cells are : *, P<0.05; **, P<0.01.
