# Supplementary material for: Essential role for SphK1/S1P signaling to regulate hypoxia-inducible factor 2α expression and activity in cancer
Source: Oncogenesis. 2016 Mar 14;5(3):e209–. doi: 10.1038/oncsis.2016.13 (PMC4815047; doi:10.1038/oncsis.2016.13)
Supplement: Supplementary Figure 2 [file oncsis201613x2.pdf]

A.

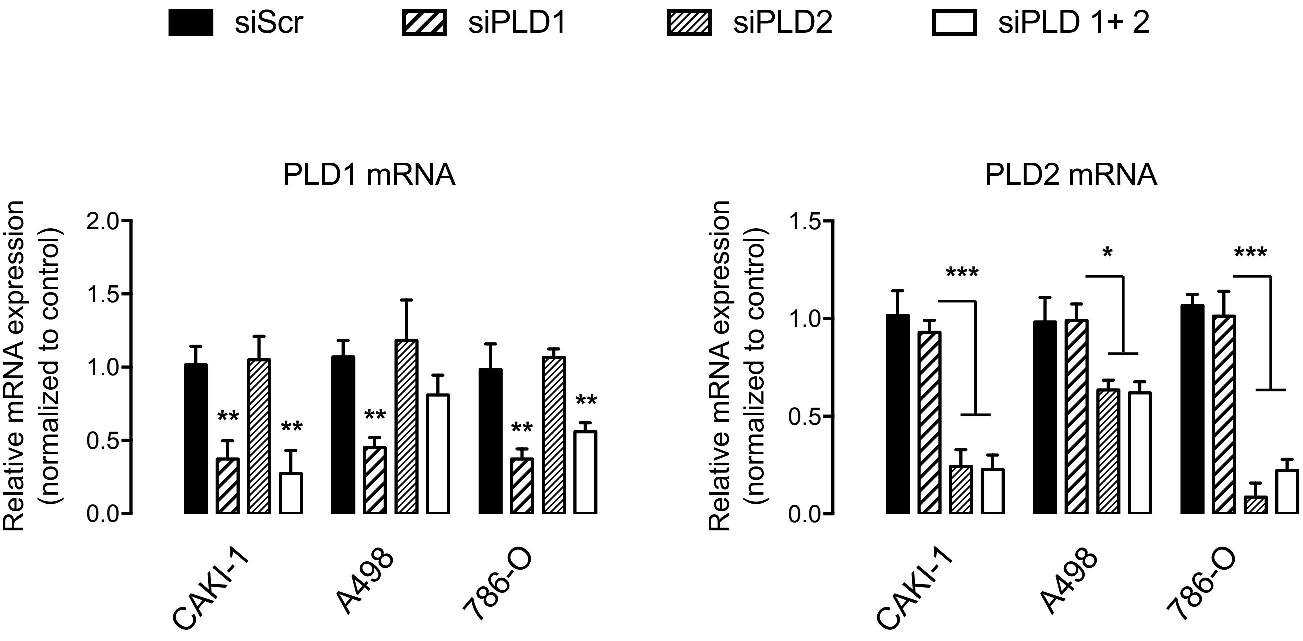

B.

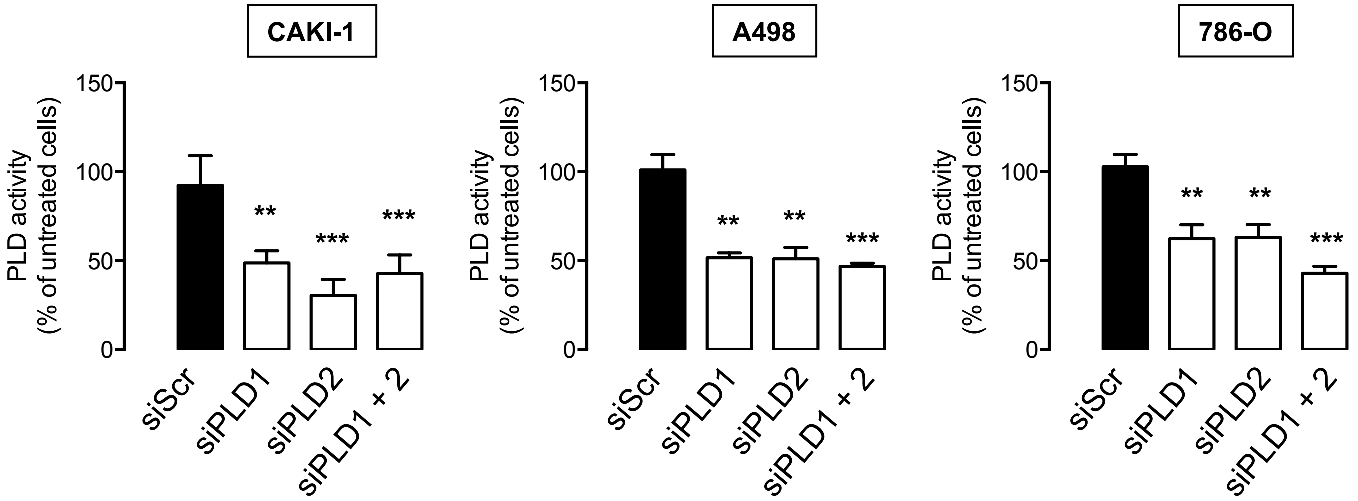

Validation of siRNA against PLD1 and 2 isoforms

CAKI-1, A498 and 786-O cells were transfected with siPLD1 (50 nmol/L), siPLD2 (50 nmol/L) or siPLD1 (50 nmol/L) and siPLD2 (50 nmol/L) or scrambled siRNA (siScr, 50 nmol/L) for 72h then assayed for mRNA expression (**A**) or enzymatic PLD activity (**B**). *Columns*, mean of at least three independent experiments; *bars*, SEM. The two-tailed P values between the means of hypoxic cells are : *, P<0.05; **, P<0.01; ***, P<0.001.
