# Supplementary material for: Essential role for SphK1/S1P signaling to regulate hypoxia-inducible factor 2α expression and activity in cancer
Source: Oncogenesis. 2016 Mar 14;5(3):e209–. doi: 10.1038/oncsis.2016.13 (PMC4815047; doi:10.1038/oncsis.2016.13)
Supplement: Supplementary Figure 3 [file oncsis201613x3.pdf]

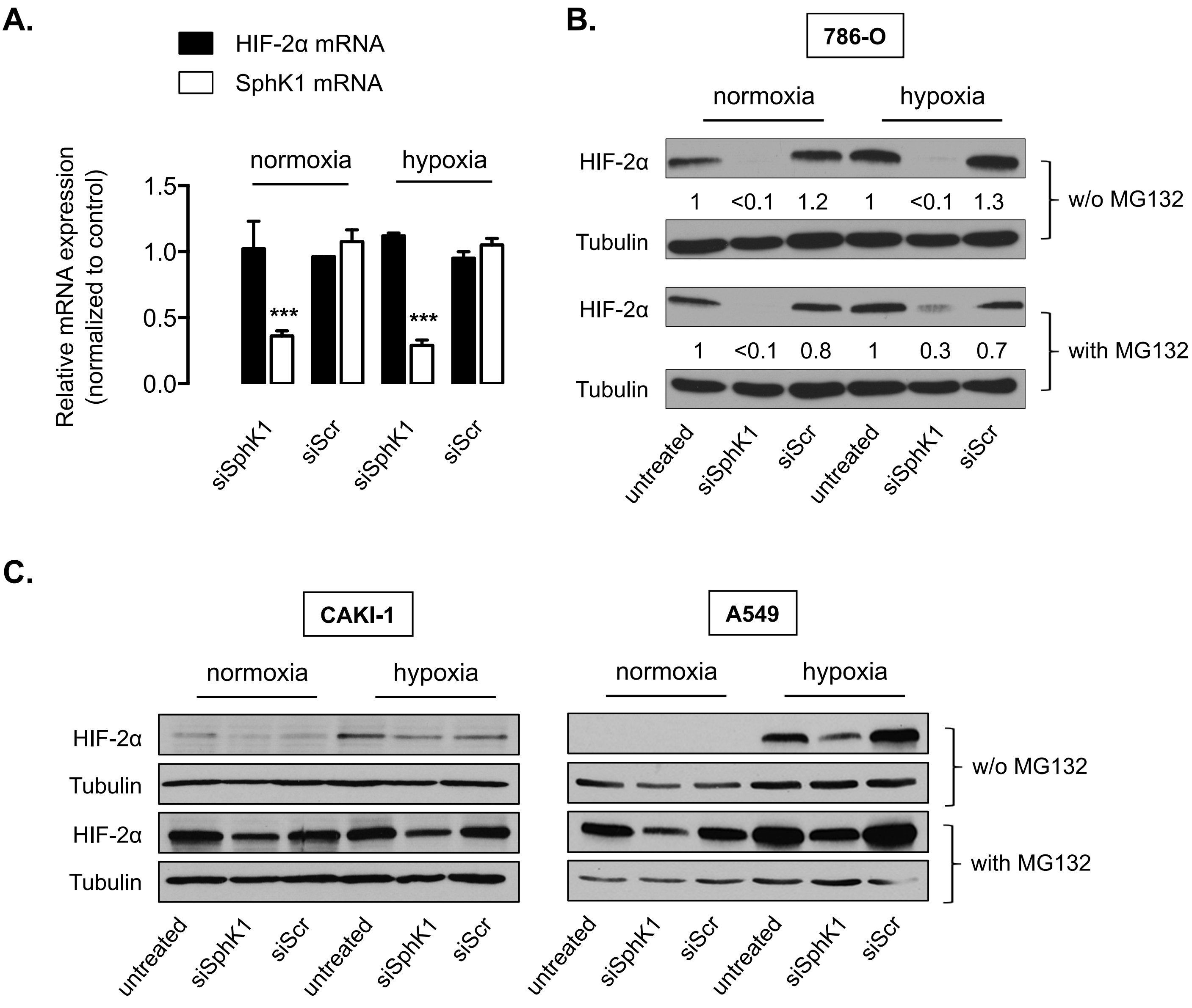

SphK1 regulates proteasome-independent HIF-2α degradation in *VHL*-defective 786-O cells in *VHL* wild-type CAKI-1 and A549 cells

A, relative mRNA expression of HIF-2α and SphK1 expression in 786-O cells was measured after 72h of treatment with 20 nmol/l of siSphK1 or scrambled siRNA (siScr) followed by 6h under normoxic or hypoxic condition. *Columns*, mean of at least four independent experiments; *bars*, SEM. ***, P<0.001. **B**, 786-O cells were untransfected or transfected with 20 nmol/l of siSphK1 or scrambled siRNA (siScr) for 72h before the experiments. Cells were then incubated for 6h under normoxic or hypoxic condition in presence or absence of the proteasome inhibitor MG132 (10 μM). Cell lysates were assayed for HIF-2α expression by Western blot analysis. Similar results were obtained in three independent experiments, and equal loading was monitored using antibody to tubulin.

C, CAKI-1 and A549 cells were untransfected or transfected with 20 nmol/l of siSphK1 or scrambled siRNA (siScr) for 72h before the experiments. Cells were then incubated for 6h under normoxic or hypoxic condition in presence or absence of the proteasome inhibitor MG132 (10 μM). Cell lysates were assayed for HIF-2α expression by Western blot analysis. Similar results were obtained in three independent experiments, and equal loading was monitored using antibody to tubulin.
