# Supplementary material for: Essential role for SphK1/S1P signaling to regulate hypoxia-inducible factor 2α expression and activity in cancer
Source: Oncogenesis. 2016 Mar 14;5(3):e209–. doi: 10.1038/oncsis.2016.13 (PMC4815047; doi:10.1038/oncsis.2016.13)
Supplement: Supplementary Figure 4 [file oncsis201613x4.pdf]

Bouquerel et al. (Suppl Fig 4)

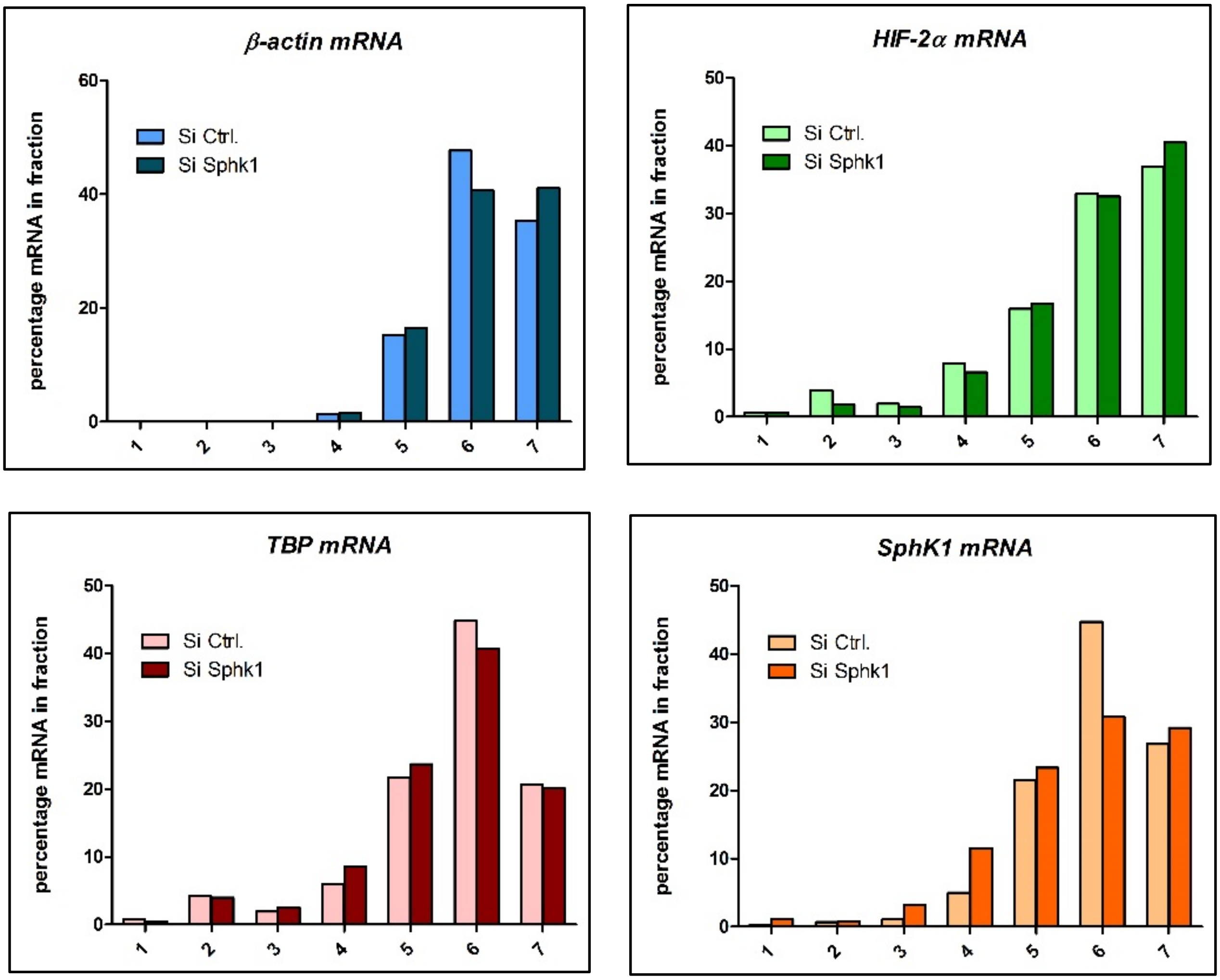

Distribution of mRNA along polysomes fractions.
RNA were extracted from fractions indicated in Fig 5D and quantified by RT-qPCR for the indicated mRNA. Results are presented as relative distribution in percent for each fraction.
