# Supplementary material for: Essential role for SphK1/S1P signaling to regulate hypoxia-inducible factor 2α expression and activity in cancer
Source: Oncogenesis. 2016 Mar 14;5(3):e209–. doi: 10.1038/oncsis.2016.13 (PMC4815047; doi:10.1038/oncsis.2016.13)
Supplement: Supplementary Figure 5 [file oncsis201613x5.pdf]

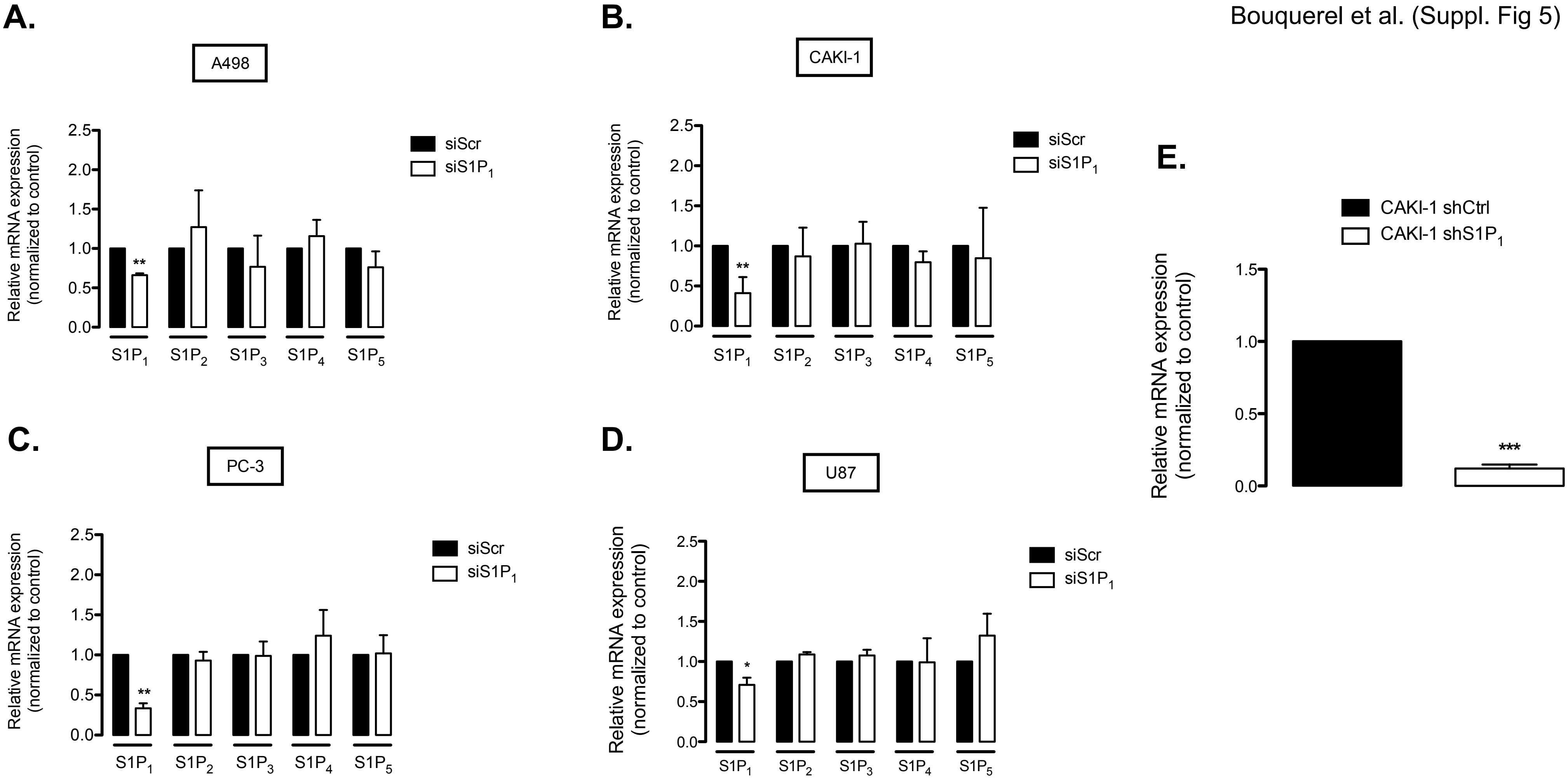

Validation of RNA interference strategies against $S1P_1$

A-D, A498 (**A**), CAKI-1 (**B**), PC-3 (**C**) and U87 (**D**) cells were transfected with 50 nmol/l of $siS1P_1$ or scrambled siRNA (siScr) for 72h, then assayed for relative $S1P_{1-5}$ mRNA expression. *Columns*, mean of at least three independent experiments; bars, SEM. *****, $P<0.05$; ******, $P<0.01$. **E**, Relative $S1P_1$ mRNA expression in CAKI-1 shCtrl (black) and CAKI-1 $shS1P_1$ (white) cells. *Columns*, mean of three independent experiments; bars, SEM. *******, $P<0.001$.
