# Supplementary material for: Essential role for SphK1/S1P signaling to regulate hypoxia-inducible factor 2α expression and activity in cancer
Source: Oncogenesis. 2016 Mar 14;5(3):e209–. doi: 10.1038/oncsis.2016.13 (PMC4815047; doi:10.1038/oncsis.2016.13)
Supplement: Supplementary Figure 6 [file oncsis201613x6.pdf]

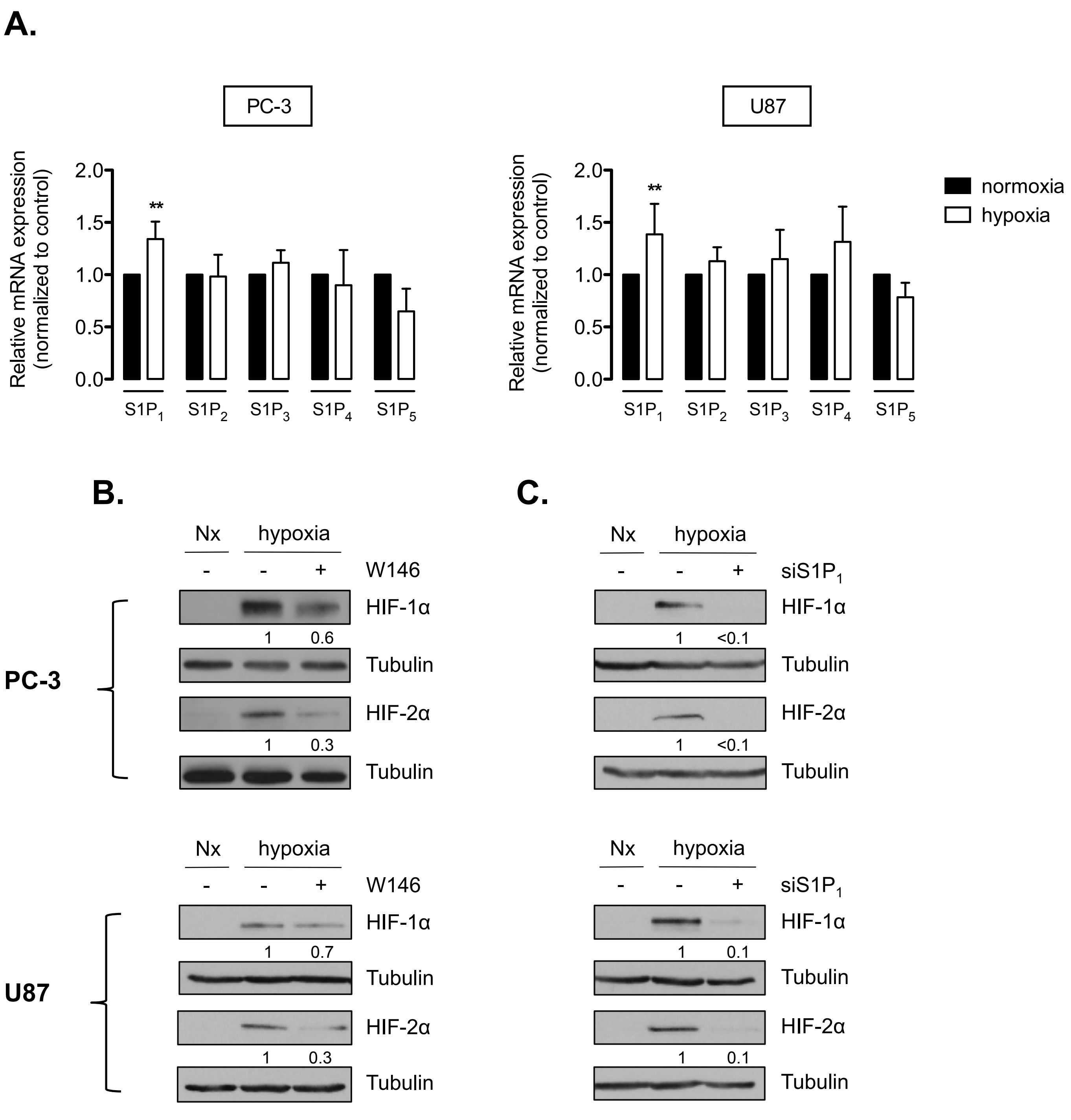

$S1P_1$ regulates both HIF-1α and HIF-2α accumulation in PC-3 and U87 cells under hypoxia
A, the relative mRNA expression of $S1P_{1-5}$ in PC-3 and U87 was measured after 1h of incubation under normoxic (black) or hypoxic (white) conditions. *Columns*, mean of at least five independent experiments; *bars*, SEM. **, P<0.01. **B**, PC-3 and U87 cells were treated with W146 (5µM) or ethanol (control), then incubated under normoxia (Nx) or hypoxia for 6h. HIF-1α and HIF-2α expression were analyzed by immunoblotting. **C**, PC-3 and U87 cells were transfected with 50 nmol/l of $siS1P_1$ or scrambled siRNA for 72h, then incubated under normoxia (Nx) or hypoxia for an additional 6h. Cell lysates were assayed for HIF-1α and HIF-2α expression by immunoblotting. For all experiments, similar results were obtained in at least three independent experiments, and equal loading was monitored using antibody to tubulin.
