# Supplementary material for: Essential role for SphK1/S1P signaling to regulate hypoxia-inducible factor 2α expression and activity in cancer
Source: Oncogenesis. 2016 Mar 14;5(3):e209–. doi: 10.1038/oncsis.2016.13 (PMC4815047; doi:10.1038/oncsis.2016.13)
Supplement: Supplementary Figure 7 [file oncsis201613x7.pdf]

A.

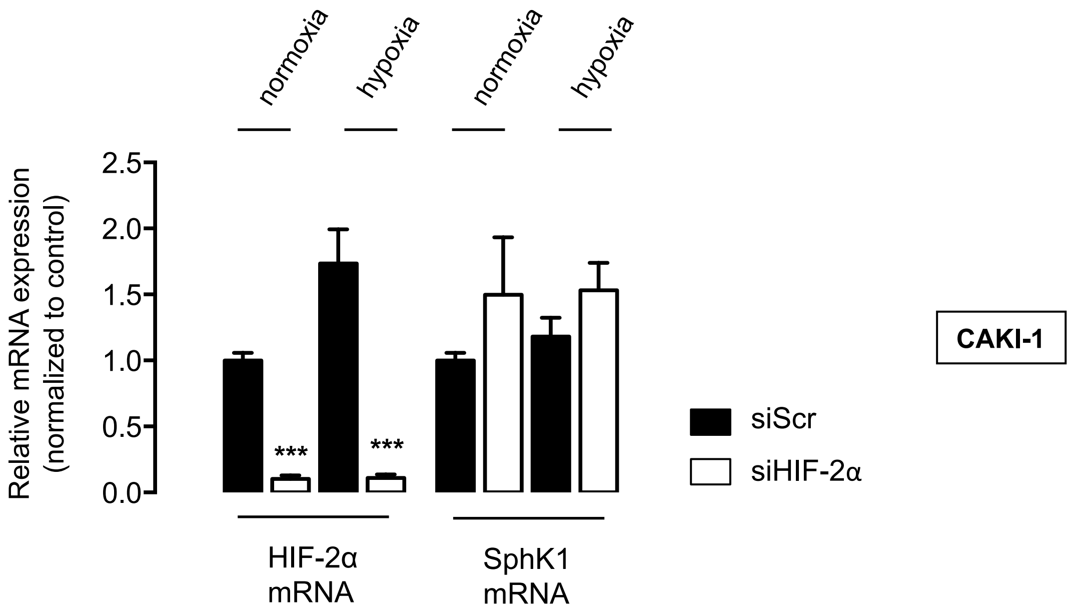

B.

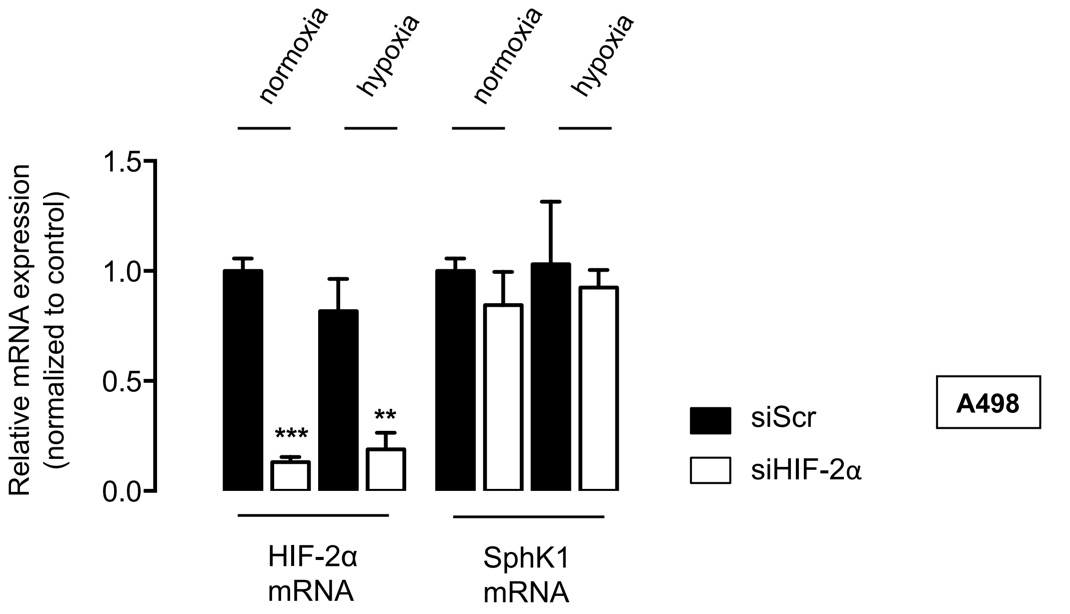

HIF-2 activity does not regulate SphK1 mRNA expression
relative mRNA expression of HIF-2α and SphK1 expression in CAKI-1 (**A**) and A498 (**B**) cells was measured after 72h of treatment with 20 nmol/l of siHIF-2α or scrambled siRNA (siScr) followed by 6h under normoxic or hypoxic condition. *Columns*, mean of at least five independent experiments; *bars*, SEM. **, $p<0.01$; ***, $p<0.001$.
